# Bayesian methods for the design and interpretation of clinical trials in very rare diseases

**DOI:** 10.1002/sim.6225

**Published:** 2014-06-23

**Authors:** Lisa V Hampson, John Whitehead, Despina Eleftheriou, Paul Brogan

**Affiliations:** a Medical and Pharmaceutical Statistics Research Unit, Department of Mathematics and Statistics, Lancaster UniversityLancaster, LA1 4YF, U.K.; b Department of Paediatric Rheumatology, UCL Institute of Child Health30 Guilford Street, London WC1N 1EH, U.K.

**Keywords:** allocation ratio, Bayesian model, expert opinion, prior elicitation, prior power, rare diseases

## Abstract

This paper considers the design and interpretation of clinical trials comparing treatments for conditions so rare that worldwide recruitment efforts are likely to yield total sample sizes of 50 or fewer, even when patients are recruited over several years. For such studies, the sample size needed to meet a conventional frequentist power requirement is clearly infeasible. Rather, the expectation of any such trial has to be limited to the generation of an improved understanding of treatment options. We propose a Bayesian approach for the conduct of rare-disease trials comparing an experimental treatment with a control where patient responses are classified as a success or failure. A systematic elicitation from clinicians of their beliefs concerning treatment efficacy is used to establish Bayesian priors for unknown model parameters. The process of determining the prior is described, including the possibility of formally considering results from related trials. As sample sizes are small, it is possible to compute all possible posterior distributions of the two success rates. A number of allocation ratios between the two treatment groups can be considered with a view to maximising the prior probability that the trial concludes recommending the new treatment when in fact it is non-inferior to control. Consideration of the extent to which opinion can be changed, even by data from the best feasible design, can help to determine whether such a trial is worthwhile. © 2014 The Authors. *Statistics in Medicine* published by John Wiley & Sons, Ltd.

## 1. Introduction

For clinical investigators working to improve the treatment of diseases with very low incidence rates, traditional sample size requirements for clinical research are daunting. Regulatory guidance on trials in small populations 1 advises that alternative approaches to the statistics of such trials might be suitable if they can improve the interpretability of trial results. Lilford *et al.* 2 discuss this problem and suggest the Bayesian approach as one suitable alternative. Billingham *et al.* 3 further highlight the advantages of the Bayesian approach for communicating the results of small trials. The posterior distribution of the treatment effect, representing the current state of knowledge after the trial has been conducted, may be useful for assessing treatment options even when the limited data available do not allow conclusive inferences to be drawn. In the Bayesian paradigm, an informative prior distribution for the unknown treatment effect can be determined from either expert knowledge or related data available at the time of planning a study. Data on controls from historical randomised controlled trials (RCTs) can be synthesised in a Bayesian random-effects meta-analysis and used to derive a prior for the parameter in the new trial accounting for both parameter uncertainty and between-trial heterogeneity 4–6. Alternatively, power priors 7 incorporate existing information by raising the likelihood of the historical data to either a fixed or unknown power to discount their contribution to prior opinion. Tan *et al.* 8 suggest down-weighting available data according to their anticipated relevance to the future study and the quality of the study from which they were generated. Clinical research in children is one setting where sample sizes available for clinical trials are limited because diseases are rare or recruitment is challenging. Authors have proposed Bayesian designs for paediatric trials, which borrow strength from adult data to reduce prior uncertainty about drug effects in children. Goodman and Sladky 9 derive an informative prior distribution for a hazard ratio in children by discounting evidence obtained from a meta-analysis of adult trials. Meanwhile, Schoenfeld *et al.* 10 use a Bayesian hierarchical model to describe data generated from adult and paediatric studies, assuming that treatment effects in the two age groups are exchangeable. When relevant historical data are not available, prior distributions must be determined from expert opinion. A rich literature has been written on methods for elicitation and the heuristics that may bias judgements of subjective probabilities 11–15. Examples of elicitation have been cited in the fields of engineering, finance and medicine, amongst others (16 and 17, Chapter 10). Chaloner and Rhame 18 highlight the benefits for frequentist clinical trials of documenting individuals’ prior beliefs because this allows assessment of whether the interim results of a trial would convince the wider clinical community to change practice. In clinical trial applications, prior opinion has been elicited through a variety of means including face-to-face meetings 19, e-mail, telephone and postal questionnaires 18,20,21. Face-to-face meetings have the advantage that training and feedback can be provided to the expert, increasing the chances that the elicited distribution is an accurate representation of their beliefs. However, the time and cost needed to engage with subject-matter experts in this way has to be balanced against the wish to elicit beliefs from a wide range of experts in order to capture the full spectrum of prior opinion. The purposes of this paper are to propose a new Bayesian framework for clinical research in very rare diseases and to illustrate how it might be used in practice. It is important at the outset to state the limits of what a Bayesian approach can achieve. If hundreds or thousands of subjects are needed to make a definitive statement according to a conventional sample size calculation, then that is what is needed. A Bayesian analysis of a smaller sample can lead to some improvement in the understanding of a treatment, but not to conclusions of comparable confidence. The methods described here represent a last resort, when satisfactory sample sizes cannot be accumulated within a reasonable period, and should not be contemplated when a conventional, high-powered trial can (perhaps with some effort, cooperation and adequate funding) be undertaken. This research was motivated by the design of the MYPAN trial, a multicentre RCT comparing mycophenolate mofetil (MMF) with cyclophosphamide (CYC) for the treatment of polyarteritis nodosa (PAN), a rare and serious inflammatory blood vessel disease in children. The primary endpoint is disease remission within 6 months of randomisation according to standard criteria. It has been estimated that a consortium of 20–30 centres from 14 European countries would recruit about 14 suitable patients per year, so that a target sample size of 40 patients is regarded as feasible. Group sequential monitoring can achieve reductions in expected sample size of up to around 40% on the fixed sample size: 22 the triangular test is approximately optimal in the sense that it minimises the maximum expected information on termination over values of the treatment effect 23, p. 79, and this design has been widely implemented in practice (e.g. www.mps-research.com/PEST). However, the benefits for early stopping of group sequential monitoring will have little impact on the feasibility of the MYPAN trial because the sample size required by a definitive fixed sample test is in the region of 383–513 patients per treatment arm. For these reasons, the MYPAN trial follows a Bayesian design. There has never been a clinical trial in children with PAN before. Treatment with CYC has been standard for the past 35 years, and although effective, it is toxic with adverse effects including excessive infection, nausea, bladder toxicity and haemorrhage, infertility and malignancy 24. MMF is a newer, orally administered immunosuppressant that is expected to have a much better toxicity profile than CYC and is likely to be almost as effective. Therefore, MYPAN is designed as a non-inferiority trial with a pre-specified non-inferiority margin of *ξ* = 0.1 on the probability difference scale. A 2-day meeting was held to elicit experts’ prior beliefs about 6-month remission rates on CYC and the relative efficacy of the trial treatments. Opinion was also sought about the relevance to the MYPAN trial of data from the MYCYC trial, a recently analysed (but unpublished) RCT comparing MMF and CYC in anti-neutrophil cytoplasmic antibody (ANCA)-associated vasculitis, a condition related to PAN. Fifteen experts from across the European Union with substantive experience of treating children with PAN participated in the meeting. This paper describes the design of the MYPAN trial and how expert opinion and related data were combined to derive consensus prior distributions. In Section 2, a Bayesian model for the MYPAN trial data is formulated, and in Sections 3 and 4, we outline how prior distributions for model parameters were determined. The statistical software used to elicit these prior distributions is also discussed. In Section 5, we consider how expert prior opinion can be used to inform trial design, choosing the treatment allocation ratio so as to maximise the probability that a trial recommends a non-inferior experimental treatment. Consideration is also given to evaluating the frequentist type I error rate of a proposed Bayesian decision rule. We conclude in Section 6 by reflecting on the implications of our experiences for the wider use of Bayesian methods to design trials in very rare diseases.

## 2. A Bayesian model

Suppose that n patients are to be recruited to the MYPAN trial, with n_E_ receiving MMF (labelled treatment E) and n_C_ receiving CYC (labelled treatment C). When the trial is complete, there will be S successes and F failures, of which S_j_ of the successes and F_j_ of the failures are on treatment j, j = E, C. Denote the probability of success on treatment j by p_j_, j = E, C. The prior knowledge about p_C_ will be expressed as a beta distribution, with parameters a and b. Let θ denote the log-odds ratio log[{p_E_(1 − p_C_)}/{p_C_(1 − p_E_)}]. The joint prior is completed by setting a distribution for θ, independently of that for p _C_. It is more likely that opinion about these two parameters will be independent than opinion about p _C_ and p _E_ will be: if p_C_ is considered to be large, then perhaps p _E_ will be thought to be slightly smaller. The prior for θ will be taken to be normal, independent of the prior for p_C_. We denote the mean and variance of this distribution by μ and σ^2^, respectively. We prefer to measure treatment effects using the log-odds ratio rather than the probability difference because it is unclear whether a normal distribution would adequately model prior opinion about a bounded quantity such as p _E_ − p_C_, which must lie between − 1 and 1. The joint prior distribution of p_C_ and θ is f_0_(p_C_,θ) = h_0_(p_C_)k_0_(θ), where 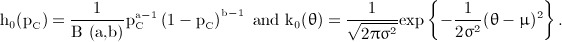


The corresponding joint prior density of p_C_ and p _E_ is (1)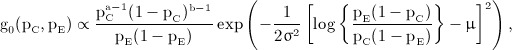 so that prior opinion about p_C_ and p_E_ is correlated, as is prior opinion about p_E_ and θ. The marginal prior density for p_E_ does not take a standard form but can be found from g_0_(p_C_,p_E_) using numerical integration. The posterior density given observed data **z** will be denoted by g(p_C_,p_E_ | **z**). It follows that 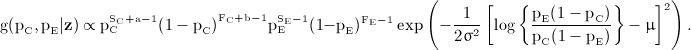


Marginal posterior distributions for p_E_,p_C_ and θ can be found using numerical integration. We have not yet explained how parameters of the prior distributions for p_C_ and θ might be determined. In Section 3, we propose an approach based on eliciting prior opinion from expert clinicians with knowledge of the condition. In Section 4, we show that this can be extended to incorporate related trial data.

## 3. Determination of a prior distribution on the basis of expert opinion

### 3.1. Eliciting expert opinion

The objective of day 1 of the MYPAN elicitation meeting was to establish prior distributions for p_E_,p_C_ and θ from expert opinion, without reference to the outcome of the MYCYC trial. For the purposes of elicitation, an ‘expert’ was defined as a paediatric consultant in rheumatology, nephrology, immunology or other allied specialism, with experience of treating on average at least one case of PAN every 2 years. Other practical considerations relevant to the conduct of the meeting are detailed in 25, including how experts were sampled and the training on Bayesian statistics they received prior to the formal elicitation exercise. Before we asked the experts for their opinions on p_C_ and θ, summaries of the current evidence for treatments in PAN, including the results of adult RCTs and low-level evidence such as case reports and retrospective case series, were presented. The proposed Bayesian model requires specification of two prior distributions, that is, a Beta(a, b) prior for p_C_ and a N(μ,σ^2^) prior for θ. Experts’ individual opinion for these parameters could be combined to derive consensus distributions using either mathematical or behavioural aggregation. Mathematical aggregation may be used to combine experts’ individual prior distributions using supra-Bayesian methods, or linear or logarithmic pooling of prior densities, where the latter approaches can accommodate unequal weightings of different experts’ opinions 17, Chapter 9. Behavioural aggregation is the process by which experts interact to reach a mutually agreeable consensus prior distribution through constructive discussions. Behavioural aggregation of opinion was preferred in this instance because participating experts had different medical specialisms, knowledge and experience of using MMF and CYC, admitting the possibility that the group would attribute unequal weights to different experts’ views in a way that would be difficult to model. Our approach was to elicit experts’ individual prior beliefs about p_C_ and θ first before asking the group to convene and reach consensus distributions through behavioural aggregation. We shall explain the process by which consensus distributions were arrived at in more detail later in this section. In order to elicit suitable parameters for the experts’ individual prior distributions, each was asked to independently complete a short questionnaire, marking responses to six questions on visual analogue scales ranging from 0 to 1, rounding probabilities to the nearest 0.05. Experts were advised that answers of 0 or 1 were not permitted to enable fitting of the parametric prior distributions in Model (1). To determine the parameters a and b, experts were asked the following:

(i) What do you think the 6-month remission rate for children with PAN treated with CYC in combination with corticosteroids (steroids) is?
(ii) Provide a proportion such that you are 75% sure that the true 6-month remission rate on CYC/steroids exceeds this value.

Question (ii) was intended to capture an individual's uncertainty about p_C_. We chose to elicit percentiles of prior distributions rather than ask, for example, ‘What is the chance that the 6-month remission rate on CYC lies between π_L_ and π_U_?’, for some choice of π_L_ and π_U_ because the latter interval may anchor answers to (i), introducing bias 17, Chapter 3. The answer to question (i) is taken as the prior mode for p_C_,(a − 1)/(a + b − 2), and the answer to (ii) as the percentile π_0.25_ such that H(π_0.25_; a, b) = 0.25, where H denotes the beta distribution function. These equations can be solved to provide values of a and b. The other two questions concerned θ and were expressed as follows:

(iii) What is the chance that the 6-month remission rate on MMF/steroids is higher than that on CYC/steroids?
(iv) What is the chance that the 6-month remission rate on CYC/steroids exceeds that on MMF/steroids by more than 10%?

**Figure 1:**
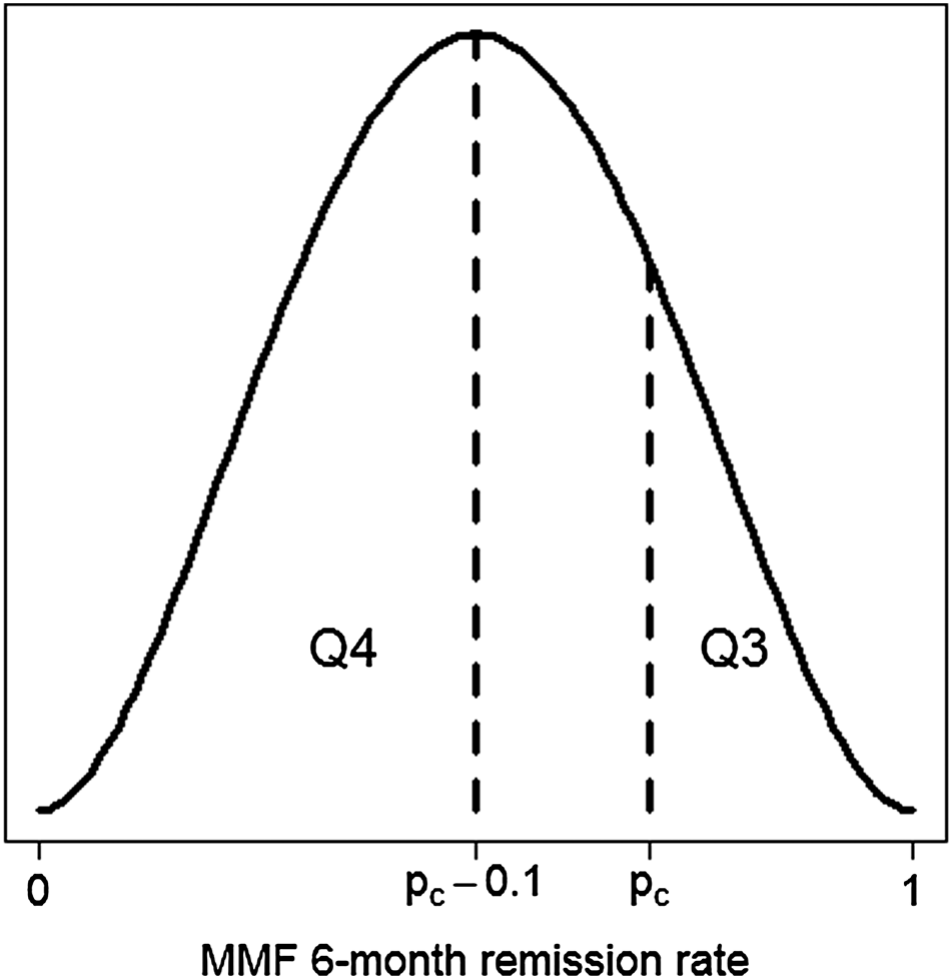
Graphical interpretation of hypothetical answers to elicitation questions (iii)–(iv).

Here, the preference of most clinicians for probability differences rather than odds ratios is acknowledged, although it would be more direct to ask the questions in terms of the latter. The answer to question (iii) can be equated to the prior probability that p_E_ > p_C_, which is Φ(μ/σ), where Φ is the distribution function of a standard normal variate. Question (iv) asks for the prior probability that p_E_ − p_C_ < − 0.1; that is, MMF is inferior to CYC by at least the pre-specified non-inferiority margin. To reflect the prior uncertainty about p_C_ and p_E_, we write this probability as an integral of the prior joint density g_0_(p_C_,p_E_). As this joint density can be expressed in terms of σ and parameters that have already been fixed by answers to questions (i)–(iii), numerical integration of g_0_(p_C_,p_E_) can be used in a univariate search to determine a suitable value of σ and hence μ. We have proposed a simple approach to determining prior distributions for the Bayesian model parameters. Although the chosen beta and normal models will not accommodate all opinion, it is unlikely that prior knowledge is so detailed that they will not provide an acceptable approximation. Graphical interpretations of hypothetical answers to questions (iii) and (iv) (as shown in Figure 1) helped to clarify experts’ understanding of the quantities sought. Figure 1 illustrates the ordering that answers should follow; that is, the answer to (iii) should be less than 1 minus the answer to (iv). Experts were also advised that the opinion that MMF and CYC would have similar efficacy could be represented by an answer to question (iii) of 0.5 and an answer to question (iv) of close to 0. It is useful to ask more questions than there are model hyperparameters to allow the model's goodness of fit to be assessed and inconsistencies in the experts’ opinions to be detected (17, Section 6.3 and 26). Therefore, each expert was asked two further questions about their opinion for p _E_, which were expressed as follows:

(v) What do you think the 6-month remission rate on MMF/steroids is?
(vi) Provide a proportion such that you are 75% sure that the true 6-month remission rate on MMF/steroids exceeds this value.

We were wary of overwhelming the experts with too many redundant questions in case this caused fatigue or unnecessary confusion.

**Figure 2:**
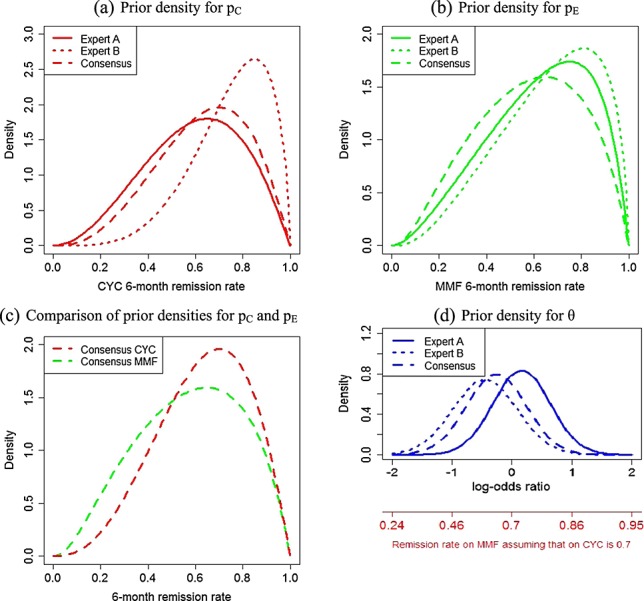
Prior densities for p_C_,p_E_ and θ elicited from two individuals (labelled experts A and B) and the densities of the consensus prior distributions agreed by the 15 participating experts as representing their collective opinion. All prior densities were elicited without reference to the MYCYC data. Expert A's distributions were defined by the following responses to the elicitation questions: (i) 0.65, (ii) 0.45, (iii) 0.63 and (iv) 0.05. Expert B's distributions were defined by the following answers: (i) 0.85, (ii) 0.65, (iii) 0.2 and (iv) 0.4.

Once each expert had completed the questionnaire, they had a one-to-one meeting with a statistical facilitator who fed back plots of the fitted probability density functions (PDFs) and summaries of the marginal prior distributions, including 90% credibility intervals, measures of location (mode and mean) and the strength of prior opinion (standard deviations and prior effective sample sizes (ESSs)). When providing feedback, emphasis was placed on the p_C_ and p_E_ prior distributions, recognising that log-odds ratios can be challenging to interpret. In an attempt to overcome these difficulties, we interpreted the PDF of θ informally in terms of a prior distribution for p_E_ assuming p_C_ is fixed at the individual's prior mode stipulated by question (i) (e.g. Figure 2(d)).

Summaries of elicited priors and PDFs were generated by user-written R 27 code calling the ‘Shiny’ package 28 to create a user-friendly interface. R routines implementing the methods described in this paper are available at http://www.research.lancs.ac.uk/portal/en/people/lisa-hampson(0d0606b8-b871-43dc-9adb-05cbb053a26e).html. Upon receiving feedback, the experts were allowed to revise their answers to (i)–(iv) until they were satisfied with the fitted PDFs as representations of their prior opinion. Redundant questions (v) and (vi) formed part of the conversations between experts and the statistical facilitators. During these conversations, properties of the fitted p_E_ distribution were compared with the experts’ initial answers to questions (v) and (vi). The inconsistencies observed during the meeting were not that great, but had they been, this would have prompted the statistical facilitators to encourage the experts to reconsider their answers to questions (i)–(iv). Only answers to the first four elicitation questions were used to estimate an expert's hyperparameters. All of the experts interviewed found a combination of answers for which the fitted distributions for p_C_,p_E_ and θ had face validity. Figure 2 shows the final prior densities for two individuals with extreme answers to questions (i)–(iv) to illustrate the range of responses recorded. Once each expert was satisfied with their prior distribution, the group reconvened to share and discuss their answers to (i)–(iv). When planning the MYPAN elicitation meeting, care was taken to set aside sufficient time for structured group discussions that followed a similar approach to the nominal group technique 29, pp. 472–474. Experts took turns to explain their answers to questions (i)–(iv) and had the option to modify them in light of the explanations and comments of others. Several of the experts did take this opportunity, and individuals’ final answers to questions (i)–(iv) are presented in 25. One of the statistical facilitators then took the mean and medians of the final individual answers to (i)–(iv) and asked the experts whether the distributions for p_C_,p_E_ and θ that these implied had face validity as their consensus priors. During this process, priors were characterised by their ‘ESSs’ 30 as a means of communicating to the experts the strength of opinion that their distributions represented. ESSs were well understood by the subject-matter experts and were influential on the group's eventual consensus answers to (i)–(iv) because the group wished to temper the strength of opinion represented by their consensus distribution for θ. In the next section, we explain how ESSs were calculated for prior distributions following Model (1).

### 3.2. Characterising expert prior opinion

When characterising their prior opinion, it is helpful for experts to be shown the implications of their answers to questions (i)–(iv) in a variety of ways. One is the straightforward display of the prior densities; another is to represent the strength of their opinion in terms of equivalent numbers of observations. To determine the ESS of a parametric prior p_0_(κ), Morita *et al.* 30 first consider the posterior that would result from combining n observations with joint density f(z_1_, …,z_*n*_; κ) with a ‘non-informative’ prior found by modifying p_0_(κ) to have a large variance. The ESS of p_0_(κ) is then defined as the sample size n* for which the corresponding posterior represents the same information for κ as p_0_(κ), taking information to be Fisher's expected information evaluated at 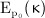. We adopt a similar approach to calculating ESSs here. For technical reasons discussed in Appendix A, we calculate the ESSs of priors k_0_(θ) and p_0_(ω), where ω = log{p_C_/(1 − p_C_)}. As these prior distributions are independent and so can be thought of as representing distinct sources of information, we calculate a prior ESS for each 30. We define the ESS of p_0_(ω) as the sample size for which the prior expected Fisher's information for ω generated by a single-arm study is equal to 1/var_0_(ω), where var_0_(ω) is the prior variance of ω, and expectations of Fisher's information are taken with respect to p_0_(ω). As θ is a measure of the advantage of E over C, we define the ESS of k _0_(θ) as the sample size for which a comparative fixed sample trial attains prior expected Fisher's information for θ equal to 1/var _0_(θ). Full details of the necessary computations are given in the Appendix.

### 3.3. MYPAN consensus prior distributions on the basis of expert opinion

Figure 2 illustrates the consensus prior distributions for p_C_,p_E_ and θ agreed by the 15 experts without knowledge of the MYCYC trial data. Prior distributions p_C_ ∼ Beta(3.6, 2.1) and θ ∼ N( − 0.26,0.25) were implied by the following responses to the elicitation questions: (i) 0.7, (ii) 0.5, (iii) 0.3 and (iv) 0.3. The answer to question (iv) implies that experts are confident that MMF is non-inferior to CYC, with a prior probability of 0.7 that 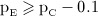. The prior for p_C_ has expectation 0.63, standard deviation 0.19 and 90% credibility interval (0.30, 0.91). The ESS of the prior for log{p_C_/(1 − p_C_)} implied by this distribution is five patients on CYC. The ESS of the prior distribution for θ is 39 patients on each treatment, and a 90% credibility interval for θ is ( − 1.09, 0.56). Under Model (1), the prior distribution for p _E_ has expectation 0.57, mode 0.65 and standard deviation 0.21. The 90% credibility interval for p _E_ is (0.21, 0.90). Prior distributions imply that experts are confident about the relative efficacies of CYC and MMF in the population eligible for the MYPAN trial, but there is greater uncertainty about absolute remission rates. Once a prior distribution has been established, and before any data are collected, the consequences of trials of various designs can be assessed. For each sample size and allocation ratio under consideration, possible values of (S_E_,F_E_,S_C_,F_C_) can be considered. The resulting posterior distributions can then be displayed. For example, during the MYPAN elicitation meeting, experts were shown how their consensus priors would be updated by datasets with 20 patients on each arm. Fixing the ‘observed’ 6-month number of remissions on CYC at S_C_ = 14, cases were considered in which the observed remission rate on MMF was equivalent, just inferior and clearly inferior to that on CYC, that is, (1) S_E_ = 14; (2) S_E_ = 12; and (3) S_E_ = 10. Such an exercise can demonstrate whether the results of a small trial will influence prior opinion enough to change clinical practice. It also provides experts with an opportunity to reflect on whether their prior distributions are overly confident. So far, we have described how prior distributions were determined on the basis of expert opinion alone. However, at the time of the MYPAN elicitation meeting, data were available from a soon-to-be-published trial. We incorporated these data into the priors, to reflect what the state of knowledge will be once they are generally known. We discuss next how this was achieved.

## 4. Determination of prior distributions combining expert opinion with historical data

### 4.1. Incorporating data from a related trial

The MYCYC trial (http://www.clinicaltrials.gov/show/NCT00414128) randomised n = 140 patients with ANCA-associated vasculitis to receive either MMF or CYC. Most trial participants were adults (66 per arm), with few aged 16 years or younger (four per arm). The primary endpoint was remission within 6 months of randomisation according to standard criteria, similar to the MYPAN trial. Data from the MYCYC trial might be considered related to the MYPAN study, although not perfectly relevant owing to differences in the populations that each study is concerned with. On day 2 of the elicitation meeting, we explored whether the MYCYC data should be incorporated into the prior distributions for p_C_, p_E_ and θ established on day 1. The next section describes how expert judgement was used to measure the relevance of these data to the planned trial.

### 4.2. Eliciting opinion on the relevance of the MYCYC data

Let p_ER_ and p_CR_ represent the 6-month remission rates on MMF and CYC in the population of which the MYCYC participants are representative. The MYCYC trial randomised n_CR_ = 70 patients to CYC and n_ER_ = 70 to MMF. Analysing the study according to the intention-to-treat principle, of those patients randomised to receive CYC, S_CR_ = 52 achieved the primary endpoint and F_CR_ = 18 did not. In the MMF group, S_ER_ = 51 and F_ER_ = 19. We link the 6-month remission probabilities in the two trial populations via the log-odds ratios 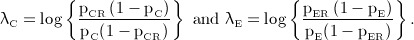


If, *a priori*, characteristics of patients recruited into the related and future trials are known to differ systematically in a way that can be predicted, it is not realistic to regard the parameters in the different trials as exchangeable 31 nor to represent their priors as independent and identically distributed. Instead, we use the link parameters λ_C_ and λ_E_ to measure differences in the effects of treatments between the two trials. Prior uncertainty about the relevance of the MYCYC data, that is, the relationships between p_C_ and p_CR_ and between p_E_ and p_ER_, is represented by the prior distributions for λ_C_ and λ_E_. This is a variation on the approach of Pocock 32 who relates control response rates in historical and contemporary trials via an additive bias parameter, updating the prior distribution for p_C_ assuming sample sizes are sufficiently large for asymptotic distributional results to apply. Suppose that independent distributions p_C_ ∼ Beta(a, b), 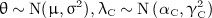 and 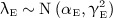 represent prior opinion about the MYPAN trial parameters without the MYCYC data, and denote the related MYCYC data by **z**_R_. On day 2 of the elicitation meeting, the design of the MYCYC trial was explained to the experts, and the baseline characteristics of the participants (demographic and clinical) were presented. Then, before revealing the trial results, experts were asked to complete a short questionnaire to elicit individuals’ beliefs about λ_C_ and λ_E_. To elicit opinion on λ_C_, experts were asked the following:

(a) What is the chance that the 6-month remission rate on CYC/steroids in the MYCYC patient group exceeds that in the MYPAN patient group?
(b) What is the chance that the 6-month remission rate on CYC/steroids in the MYPAN patient group exceeds that in the MYCYC patient group by more than 10%?Two similar questions, framed in terms of MMF/steroids, were asked to elicit opinion for λ_E_
(c) What is the chance that the 6-month remission rate on MMF/steroids in the MYCYC patient group exceeds that in the MYPAN patient group?
(d) What is the chance that the 6-month remission rate on MMF/steroids in the MYPAN patient group exceeds that in the MYCYC patient group by more than 10%?

We sought to elicit opinion about the similarity of response probabilities on each treatment in the different trials rather than the similarity of treatment effects, as we anticipated that it would be challenging to elicit opinion for a comparison of log-odds ratios. Questions (a) and (b) are similar in form to questions (iii) and (iv) used to elicit beliefs about θ, and the numerical routines described in Section 3.1 were used to find values of *α*_C_ and 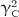 for which the fitted prior normal distribution for *λ*_*C*_ had the required properties. Graphics similar to Figure 1 were used to illustrate hypothetical answers to the elicitation questions. Experts were informed that answers to questions (a) and (b) of 0.5 would express extreme prior uncertainty about the relevance of the MYCYC data on CYC for informing opinion about CYC remission rates in the MYPAN patient group. After completion of the questionnaire, the experts reconvened to reach a group consensus for answers to questions (a)–(d), after which the MYCYC data were revealed. The day 1 prior densities for p_C_,p_E_ and θ were then updated to incorporate this new information and presented to the group. We explain in the next section how the updated prior distributions were derived.

### 4.3. Updating prior distributions to incorporate data from the MYCYC trial

**Figure 3:**
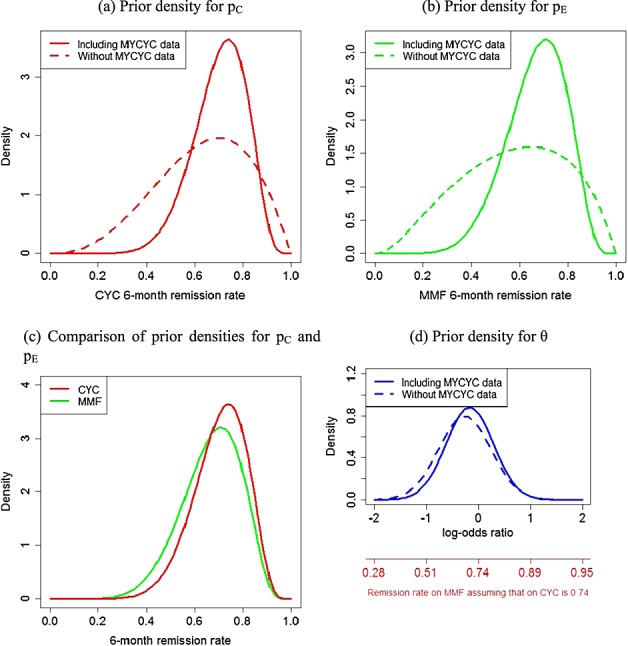
Consensus prior densities for p_C_,p_E_ and θ incorporating the MYCYC data.

The joint prior density for p_CR_, p_C_,p_ER_ and p_E_, denoted by *ϕ*_0_(p_C_,p_E_,p_CR_,p_ER_), is 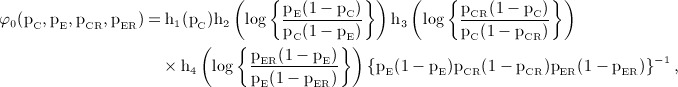


where h_1_(y),h_2_(y),h_3_(y) and h_4_(y) are the densities of Beta(a, b), 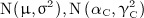 and 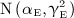 random variables, respectively, evaluated at y. The link parameters introduce correlations between p_CR_ and p_C_ and p_ER_ and p_E_ so that under the proposed model, the related data help us learn about remission rates in both trials. However, because there is uncertainty about the precise relationships between parameters in each trial, the related data are discounted for learning about p_C_ and p_E_. Using Bayes theorem to combine prior beliefs with the related data, we obtain the joint distribution for p_E_ and p_C_ as (2)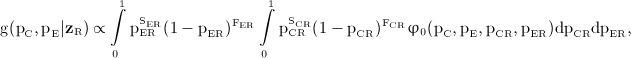 capturing the state of knowledge about p_C_ and p_E_ before the MYPAN trial is conducted. The marginal prior density for θ can be found by applying a transformation of variables to Equation (2). Prior distributions for parameters incorporating the MYCYC data are not of standard forms. Figure 3 shows densities for p_C_, p_E_ and θ that result from updating prior distributions p_C_ ∼ Beta(3.6, 2.1) and θ ∼ N( − 0.26,0.25) with the MYCYC data for the consensus responses to questions about λ_C_ and λ_E_, which were as follows: (a) 0.55, (b) 0.25, (c) 0.5 and (d) 0.25. According to these responses, λ_C_ ∼ N(0.12, 0.86) and λ_E_ ∼ N(0,0.60), consistent with the opinion that remission rates on CYC might be slightly higher in adults with ANCA-associated vasculitis than in children with PAN but that remission rates on MMF would be similar in these two populations. Incorporating the MYCYC data shifts the location of the prior for p_C_ only slightly, as the MYCYC remission rate 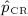 is similar to the mode of the prior elicited without reference to these data. The updated prior for p_C_ has mode 0.74, mean 0.70 and standard deviation 0.11. The location of the prior for p_E_ increases upon inclusion of the MYCYC data as 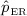 is larger than the mode of the day 1 prior, and expert opinion indicates that remission rates in the MYCYC and MYPAN patient groups are likely to be similar. The updated prior for p_E_ has mode 0.71, mean 0.67 and standard deviation 0.12. Incorporating the MYCYC data has clearly reduced uncertainty about the absolute remission rates on the two treatments: 90% credibility intervals for p_E_ and p_C_ are (0.45, 0.85) and (0.51, 0.86), respectively. The related data have less impact on the prior for θ because beliefs about θ were already rather precise before their inclusion.

**Figure 4:**
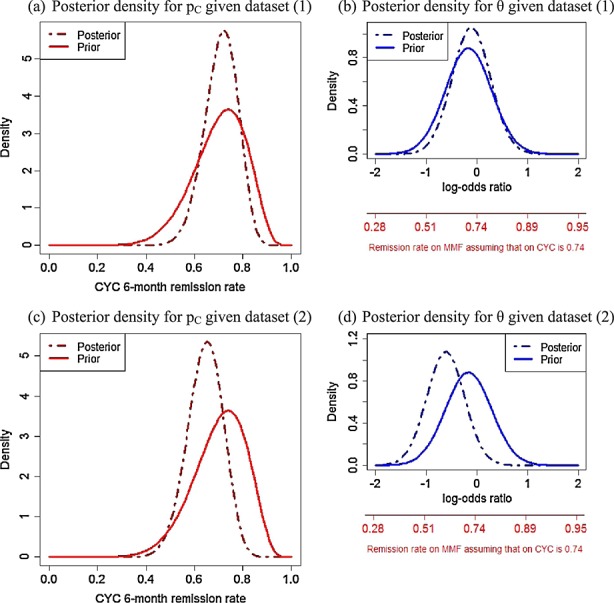
Posterior densities for p_C_ and θ given two hypothetical datasets: (1) (S_E_ = 14,F_E_ = 6,S_C_ = 14, F_C_ = 6); (2)(S_E_ = 7,F_E_ = 13,S_C_ = 14,F_C_ = 6).

The face validity of the prior densities in Figure 3 as representations of the group's beliefs after learning the MYCYC data was reviewed. Prior ESSs were presented, calculated using a similar approach to that described in the Appendix. Uncertainty about the relevance of the MYCYC data for learning about p_C_ and p _E_ means that their contribution to the prior distributions of p_C_ and θ is discounted. The ESS of the prior for log{ p_C_/( 1 − p_C_)} incorporating the MYCYC data is 17 patients, whereas for θ, the prior ESS is 48 patients on MMF and CYC. Comparing these with the ESSs of the day 1 priors, we see that the 70 MYCYC observations on MMF and CYC have been discounted to 12 and 9 observations for log{ p_C_/( 1 − p_C_)} and θ, respectively. Experts were given the option to discard the MYCYC data entirely from their prior distributions, but they chose to retain it. The posterior densities that would result from observing (S _E_ = 14, F _E_ = 6, S _C_ = 14, F _C_ = 6), (S _E_ = 7, F _E_ = 13, S _C_ = 14, F _C_ = 6) and (S _E_ = 7, F _E_ = 3, S _C_ = 7, F _C_ = 3) were presented to the experts to show the impact of hypothetical datasets on their priors. Figure 4 shows posteriors for two of these datasets. The meeting concluded with the experts agreeing to adopt the prior distributions for p_E_,p_C_ and θ shown in Figure 3 as their consensus priors for the MYPAN trial.

### 4.4. Impact of alternative priors for the relevance of the MYCYC data

In order to further explore the role of the related data, we now speculate what the impact of the MYCYC data would have been had different answers to questions (a)–(d) been elicited. First, consider what the distributions of p_E_,p_C_ and θ would have been had vague priors been specified for λ_C_ and λ_E_ by the following answers: (a) 0.5, (b) 0.45, (c) 0.5 and (d) 0.45. Then the ESSs of the prior distributions incorporating the MYCYC data would be five patients for log{p_C_/(1 − p_C_)}and 40 patients on MMF and CYC for θ. Increased uncertainty about the relevance of the MYCYC data has led to these data being discounted almost entirely, and densities for p_C_,p_E_ and θ are indistinguishable from the prior densities excluding these data. Alternatively, suppose experts had been more confident that p_CR_ exceeds p_C_ and answered (a) 0.65, (b) 0.1, (c) 0.5 and (d) 0.25, stipulating priors λ_C_ ∼ N(0.21,0.30) and λ_E_ ∼ N(0,0.60). In this setting, the prior for p_E_ would have mode 0.72 and mean 0.70, whereas the prior for p_E_ would have mode 0.70 and mean 0.66. Varying the elicitation answers to (a) 0.2 and (b) 0.5 but leaving (c) and (d) unchanged would imply λ_C_ ∼ N( − 0.51,0.37) and λ_E_ ∼ N(0,0.60), suggesting that experts were more confident that p_C_ exceeds p_CR_. Then, the prior for p_C_ would have mode 0.80 and mean 0.77, and the prior for p_E_ would have mode 0.76 and mean 0.72. We now have a framework for representing prior knowledge about treatments E and C, which can be updated once data from the future trial become available. Although trials in very rare diseases are unlikely to generate definitive levels of evidence, we recognise that decisions may need to be made on the basis of posterior distributions, such as whether continued use of E should be permitted or whether a new medicine should be licensed. In the next section, we suggest some decision criteria and explore the impact of design choices on test properties.

## 5. Choice of an allocation ratio and Bayesian decision criterion

When designing a rare-disease trial, it may be optimal in terms of power to deviate from randomising equal numbers of patients to treatments E and C if relatively little is known about p_E_. Pocock 32 chooses the optimal E:C allocation ratio in the presence of historical controls to minimise the posterior variance of a probability difference. Note that equal sample sizes were stipulated for the MYPAN trial, but we illustrate here how our methodology could be expanded to allow selection of an optimal E:C allocation ratio. For any given allocation ratio and decision criterion, all of the trial datasets that would lead to recommendation of E can be identified, and thus exact Bayesian and frequentist properties of the Bayesian procedure can be computed. We explain these calculations in the next section for a non-inferiority trial, although versions for superiority trials are straightforward.

When designing a rare-disease trial, it may be optimal in terms of power to deviate from randomising equal numbers of patients to treatments E and C if relatively little is known about p_E_. Pocock 32 chooses the optimal E:C allocation ratio in the presence of historical controls to minimise the posterior variance of a probability difference. Note that equal sample sizes were stipulated for the MYPAN trial, but we illustrate here how our methodology could be expanded to allow selection of an optimal E:C allocation ratio. For any given allocation ratio and decision criterion, all of the trial datasets that would lead to recommendation of E can be identified, and thus exact Bayesian and frequentist properties of the Bayesian procedure can be computed. We explain these calculations in the next section for a non-inferiority trial, although versions for superiority trials are straightforward.

### 5.1. Identifying the optimal E:C allocation ratio

For a Bayesian non-inferiority trial, two quantities will be of particular interest for interpreting the prior and summarising the posterior. These are Γ = P(p_E_ > p_C_) and Π = P(p_E_ > p_C_ − ξ). The prior values of these quantities, denoted by Γ_0_ and Π_0_, are respectively the answer to question (iii) and the complement of the answer to question (iv) in Section 3.1. Following 33, the Bayesian trial could be formulated so that E would be recommended as non-inferior to C if and only if the posterior value of Π exceeds some large value *ν*. We may regret not recommending treatment E when Π does not meet this threshold but Γ is promisingly large. Thus, the E:C allocation ratio could be chosen with a view to minimising Γ*, defined as the maximum value of Γ attained across the set of data quadruples (S_E_,F_E_,S_C_,F_C_) for which the posterior quantity Π *≤ ν*. That is, Γ* is the maximum value of Γ with which the trial can terminate without recommending E. Once the decision criterion for the trial has been fixed, one can evaluate the probability that E will be correctly recommended. We follow the method in 10 to define the Bayesian prior power of a non-inferiority trial as P_0_(Π > *ν* | p_E_ – p_C_ > − ξ), which, fixing n and the E:C allocation ratio, can be written as (3)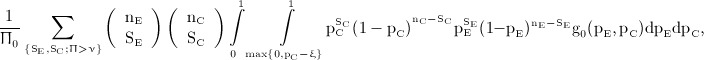 where g_0_(p_C_,p_E_) is the joint prior density (possibly conditioning on related data). Frequentist properties of the Bayesian procedure, such as the type I error rate, are also likely to be of interest even if there is no formal requirement that the design be calibrated to maintain control of this at a nominal significance level. For any combination of values for p_E_ and p_C_, the exact frequentist probability of claiming treatment E as non-inferior to C is 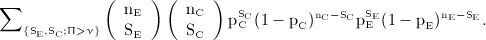


**Figure 5:**
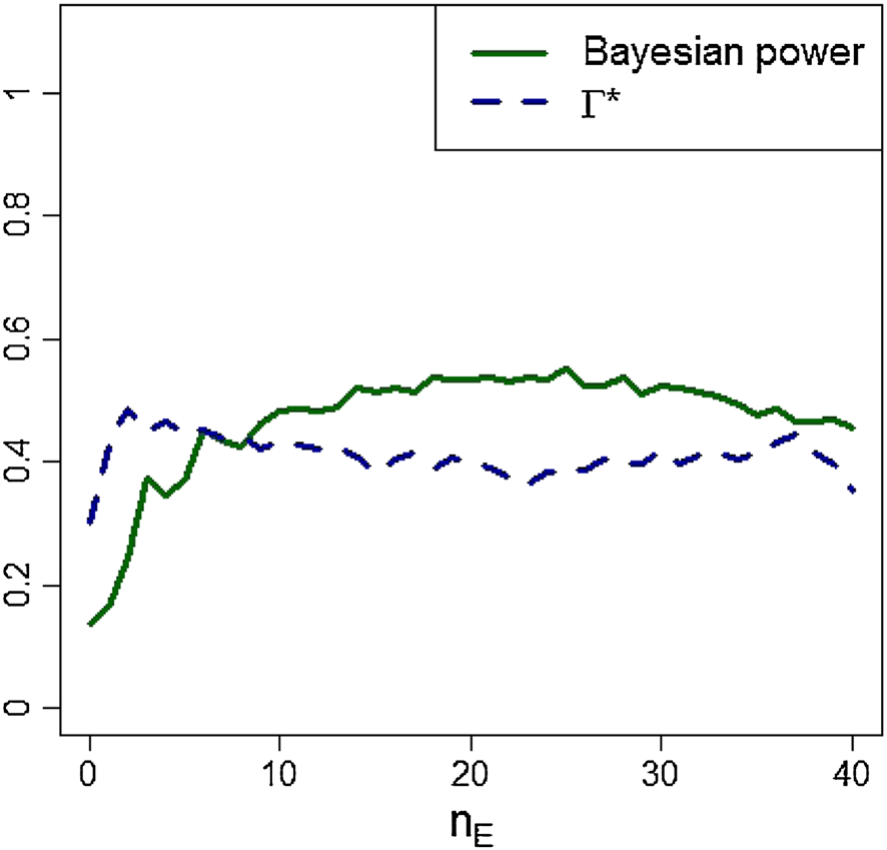
Operating characteristics of the Bayesian procedure for a range of randomisation strategies assuming prior distributions p_C_ ∼ Beta(3.6,2.1) and θ ∼ N( − 0.26,0.25). All trials have total sample size n = 40 and recommend E over C if Π > 0.8, with ξ = 0.1.

The frequentist type I error rate does not take into account the prior probability that treatment E is inferior to C. Returning to our Bayesian model, suppose treatment E will be recommended as non-inferior to C if Π > 0.8 with *ξ* = 0.1. Given this decision criterion and a sample size of n = 40, we seek the E:C allocation ratio that balances the twin objectives of attaining a high Bayesian prior power and a low value of Γ^*^. Figure 5 compares a range of designs for these criteria, taking as prior distributions p_C_ ∼ Beta(3.6, 2.1) and θ ∼ N( − 0.26,0.25). Curves for both Bayesian prior power and Γ^*^ plateau fairly quickly as n_E_ increases, meaning that there are a number of designs with good operating characteristics from which investigators can choose. For n = 40, Bayesian power is maximised at 0.55 by randomising n_E_ = 25 patients to E and n_C_ = 15 patients to C, for which design Γ^*^ = 0.38, and the frequentist type I error rate is 0.26 under p_E_ = 0.6 and p_C_ = 0.7. This value of Γ^*^ is close to the global minimum of 0.30 achieved by setting n_E_ = 0 and n_C_ = 40, for which design the Bayesian power is 0.14.

The MYPAN trial will randomise equal numbers to MMF and CYC. Assuming n = 40 and prior distributions for p_C_,p_E_ and θ are as derived in Section 4.3, the Bayesian decision rule that recommends MMF as non-inferior to CYC if Π > 0.8 has a frequentist type I error rate of 0.29 under p_E_ = 0.6 and p_C_ = 0.7. The Bayesian prior power of this design is 0.62 and Γ^*^ = 0.38. The frequentist type I error rate is high because prior opinion is confident that MMF is non-inferior to CYC by the specified margin; indeed, the prior distributions stipulate that P{p_E_ > p_C_ − 0.1} = 0.77. This optimism is little diluted by the information gathered from 40 patients. Fixing a trial's maximum sample size, E:C allocation ratio and decision criterion, it is also of interest to find the outcome 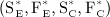 associated with the minimum value of Π for which the trial can terminate recommending E as non-inferior to C. This ‘worst-case’ dataset could be presented during the elicitation process, and experts asked whether they would be happy to recommend E as non-inferior to C on the basis of these data and their prior opinion; disagreements would lead to revisions of the prior distributions. While we did not present this information during the MYPAN prior elicitation meeting, for the MYPAN trial design with n = 40, this worst-case outcome is 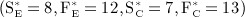 for which Π = 0.80.

## 6. Discussion

This paper describes a Bayesian framework for designing and interpreting clinical trials conducted with limited sample sizes. Prior information on Bayesian model parameters is summarised by prior distributions determined either from expert opinion or a combination of opinion and related data. Such data could be in the form of data generated in a related population, as for the MYPAN trial, or more generally data on a related endpoint or drug with a similar mechanism of action to the new medicine. One special feature of MYPAN was that the MYCYC data were genuinely unknown to the experts prior to the elicitation meeting, which enabled us to distinguish the contribution of these data from the rest of the prior opinion. This scenario may arise in other contexts such as paediatric drug development. It is a common regulatory requirement that studies supporting the development of medicines for children should follow a prospectively agreed paediatric investigation plan (PIP), prepared in the early phases of the adult development programme. In this setting, prior distributions for parameters linking success rates in adults and children could be pre-specified in the PIP before adult efficacy studies have been completed.

For the MYPAN trial, prior opinion was represented by PDFs. However, other approaches have been used elsewhere to summarise prior beliefs. For example, in the context of early-phase dose-escalation trials, Whitehead and co-authors 34,35 directly represent prior opinion about dose–toxicity and dose–exposure relationships as hypothetical observations on ‘pseudo-subjects’. In Section 4, incorporating historical data leads to prior distributions for p_C_ and p_E_ of non-standard forms. A drawback of the proposed approach when priors incorporate opinion and related data is that no simple representation of the prior distributions for p_C_,p_E_ and θ is possible. Instead, one must refer back to the consensus answers to eight elicitation questions and run numerical integration routines to obtain the needed prior densities. Dalal and Hall 36 show that the prior distribution for a single parameter can be approximated by a mixture of conjugate priors, and Schmidli *et al.* 37 use this approach to represent the prior of a response probability as a mixture of beta distributions. Further work would explore whether this approach can be extended to the two-sample comparison problem considered here, where the challenge is how to represent the joint prior distribution of p_C_ and p_E_ in a suitable conjugate form capturing correlated opinions about these two parameters.

The proposed Bayesian Model (1) does not adjust for any covariates, and this was regarded as appropriate for the MYPAN trial because randomisation will be stratified to ensure groups are balanced for key prognostic factors. The primary non-inferiority hypothesis of the trial is based on an assumption that MMF has a better side-effect profile than CYC, and to verify this, adverse events between groups will be compared as a secondary analysis to the primary efficacy analysis. In Section 5, we explored the advantages of deviating from an equal randomisation ratio. However, equal allocation between CYC and MMF is stipulated for the MYPAN trial. This is because MYPAN will be the first RCT in children with PAN: estimating remission rates on both treatments is of interest to clinicians because neither treatment has been scrutinised before in an RCT, despite the fact that CYC is the current standard of care for childhood PAN. Thus, when planning the MYPAN trial, clinicians were keen that MYPAN should contribute information for estimating absolute remission rates on MMF and CYC as well as increase understanding of the relative benefits of these treatments.

Funding of a rare-disease trial may be dependent on a prior elicitation exercise demonstrating clinically relevant levels of uncertainty about treatment effects. For example, for MYPAN, the funder (Arthritis Research UK) adopted a two-tranche funding allocation process, providing initial funds for the elicitation of prior opinion and other preparatory work and releasing the second tranche of funding to undertake the trial only if prior opinion supported this. From a charitable funding perspective, this approach is sensible because it avoids the risk of wasting money on a fruitless trial that is unlikely to generate a posterior opinion that would influence the wider clinical community. While in this paper, we have informally considered whether hypothetical data could shift prior opinion, elicited prior distributions could be used more formally in Bayesian decision theoretic analyses, such as expected value of sample information calculations 38, Chapter 12 to determine whether a small trial is worthwhile. Acceptance of prior distributions by the clinical community will be important if posterior recommendations are to change practice because in trials of very rare diseases, sample sizes will not be large enough to dilute strong prior opinion. Regulators are cautious about using Bayesian methods with informative priors to support new drug applications 1. However, the objective of many public sector clinical trials comparing licensed medicines or non-drug interventions is to improve the evidence base for treatment choices already faced by doctors and their patients. Our experiences described here demonstrate that it is feasible to elicit prior opinion to inform the design and decision to conduct an RCT in a very rare disease.

## Appendix A Deriving the effective sample size of the prior distribution of the log-odds ω

For our Bayesian model, we calculate ESSs, taking the prior information for each parameter to be the precision of the prior distribution 4. This will be an approximation unless priors follow normal distributions. Therefore, rather than calculate the ESS of the Beta(a, b) prior for p_C_, we determine the prior ESS of ω = log{p_C_/(1 − p_C_)}, the log-odds of success on C, for which a normal approximation will be more accurate. By approximating information by precision, we may determine the ESS of a prior distribution that cannot be differentiated analytically: this will be the case when priors incorporate related data. Let p_0_(ω) = exp(aω)/[B(a, b){1 + exp(ω)}^a+b^] denote the prior density of ω when p_C_ ∼ Beta(a, b), which has precision 1/var_0_(ω). We define the appropriate quantity for comparison with 1/var_0_(ω) as the prior expected information for ω that would be generated by conducting a single-arm trial treating n patients with C. With this in mind, let V_ω_(n) represent Fisher's expected information for ω that would be accrued from conducting a single-arm trial when patient responses are distributed as Y _Ci_ ∼ Bernoulli (p_C_), for i = 1, …, n. Then, V_ω_(n) = nexp(ω)/{1 + exp(ω)}^2^, and the prior expectation of V_ω_(n), denoted by E_0_[V_ω_(n)], is found by integrating information over the prior density of ω. We define the ESS of p_0_(ω) as the sample size  for which  equals the precision 1/var_0_(ω); that is,  is found satisfying

## Appendix B Deriving the effective sample size of *k*_0_(θ)

The ESS of k_0_(θ) is found using a similar approach to that described in Appendix A. The information for θ represented by prior distribution N(μ,σ^2^) is exactly σ^− 2^. Because θ is a treatment effect parameter, the appropriate quantity for comparison with 1/var_0_(θ) is the prior expected information for θ that would be generated by a two-arm trial comparing E with C. Let V_θ_(n) denote Fisher's expected information for θ that would be accumulated by an RCT with total sample size n, assuming equal allocation between E and C and that patient responses are distributed as Y_Ei_ ∼ Bernoulli(p_E_) on E and Y_Cj_ ∼ Bernoulli (p_C_) on C, for i, j = 1, …, n/2. In this setting,  23, Section 3.2, where  is the average response probability under equal allocation. Because information is a function of p_E_ and p_C_, the prior expected information, E_0_[V_θ_(n)], is found by integrating V_θ_(n) over the prior joint density of (p_E_,p_C_). We define the prior ESS of k_0_(θ) as the sample size  such that 1/var _0_( θ) is equal to ; that is,  is found satisfying
